# Abstracts/Sommaires/Zusammenfassungen

**DOI:** 10.1155/S1463924681000023

**Published:** 1981


### Abstracts Sommaires Zusammenfassungen

### Page 9

**Determination of the ultraviolet and visible spectral response of a charge-injection device array detector**

H.A. Lewis and M.B. Denton

The potential of charge-injection device (CID) electronic cameras as multichannel spectroscopic detectors is discussed. Data is presented giving the UV/visible spectral response of five CID sensors. This data shows that these particular sensors have the ultraviolet response necessary for their application as detectors in elemental analysis.

**Détermination de la réponse spectrale ultraviolette et visible d'un "charge injection device array detector" (CID)**

Le potentiel d'une caméra électronique avec "charge injection device" comme détecteur spectroscopique à multiples conduits est discuté. Les résultats sont présentés en donnant la réponse UV/VIS de cinq senseurs CID. Ces données montrent que ces senseurs ont une réponse dans l'ultraviolet nécessaire pour leur application comme détecteurs pour l'analyse élémentaire.

**Bestimmung der Spektral-Empfindlichkeit eines "Charge-Injection Device Array Detector" im ultravioletten und sichtbaren Bereich**

Es wird das Potential von elektronischen Kameras auf der Basis von "Charge-Injection Device" (CID) für spektroskopische Vielkanal-Detektoren diskutiert. Es werden Daten angeführt für die spektrale Empfindlichkeit von fünf CID Sensoren im ultravioletten und sichtbaren Bereich. Diese Daten zeigen, dass diese speziellen Sensoren die nötige ultraviolette Empfindlichkeit für ihre Applikation als Detektoren bei der elementellen Analyse aufweisen.

### Page 13

**Data-processing routines for automated relaxation times and nuclear Overhauser effect measurements on a FT NMR spectrometer**

J.P. Lemaire, B. Tiffon and B. Ancian

Fast two- and three-parameter exponential least-squares-fit routines are described. They are designed for small FT NMR spectrometer computers to calculate relaxation times from pulsed NMR experiments. A line area computation routine to increase accuracy of NOE measurements is also described.

**Evaluation de données pour mesure automatique de temps de relaxation et d'effets "Nuclear Overhouser" avec un spectromètre FT RMN**

Des méthodes rapides à deux ou trois paramètres pour régression exponentielle (least square fit) sont décrites. Elles sont prevues pour des petits spectromètres FT RMN pour calculer les temps de relaxation d'experiences par RMN pulsés. Une méthode d'évaluation de surfaces pour augmenter la precision de mesures NOE est également décrite.

**Datenverarbeitungsprogramme für die automatische Messung von Relaxationszeiten und Kernspin-Overhauser Effekt auf einem FT NMR Spektrometer**

Es werden schnelle zwei – und drei-Parameter Programme beschrieben für exponentielle Kurvenangleichung nach kleinsten Quadraten. Sie sind für die Rechner kleiner FT NMR Spektrometer konzipiert, um die Relaxationszeiten aus NMR Puls-Experimenten zu berechnen. Ein Linien-Flächenberechnungsprogramm zur Erhöhung der Genauigkeit der NOE Messungen wird ebenfalls beschrieben.

### Page 18

**A technical evaluation of the Instrumentation Laboratory Multistat I analyser**

J.F. Stevens, D.J. Allan, D.A. Rickwood and B.P. Ager

The Multistat I is an electrolyte analyser with a dedicated computer designed for the medium sized hospital laboratory. Analytical channels are provided for sodium, potassium, chloride, total $CO_2$, urea, creatinine and glucose. The instrument has been evaluated in a hospital laboratory using an accepted protocol and was generally found to be satisfactory.

**Evaluation technique de l'analyseur Instrumentation Laboratory Multistat I**

Le Multistat I est un analyseur pour électrolytes avec ordinateur dédié pour le laboratoire d'hôpital de taille moyenne. Des conduits analytiques sont prévus pour le sodium, le potassium, le chlorure, $CO_2$ total, l'uréa, la créatinine et la glucose. L'instrument a été evalué dans un laboratoire d'hôpital en utilisant un protocol accepté, et a donné des résultats satisfaisants.

**Eine technische Beurteilung des "Instrumentation Laboratory Multistat I" Analysators**

Der "Multistat I" ist ein Elektrolyten-Analysator mit permanent eingesetztem Rechner, der für das Spitallabor mittlerer Grösse konzipiert wurde. Analytische Kanäle sind vorgesehen für Natrium, Kalium, Chlorid, Gesamt-$CO_2$, Harnstoff, Kreatinin und Glucose. Das Instrument wurde in einem Spitallabor beurteilt, wobei eine anerkannte Vorschrift verwendet wurde, und wurde generell als zufriedenstellend befunden.

### Page 23

**Multilevel analysis of variance used to partition components of optical imprecision in an analyser system with disposable cuvettes**

D.M. Fast, E.J. Sampson and C.A. Burtis

The optical imprecision of equilibrium absorbance measurements on a centrifugal analyser which uses disposable cuvettes was partitioned into four components by a multilevel nested analysis of variance technique. The overall coefficient of variation for 2052 absorbance measurements on three disposable rotors from each of three lots of rotors was 3.4% ($\bar{x}$ = 0.4144). Most of the variance (68.6%) was accounted for by between-lot variation. Between rotor variation was 10.1% of the total, between-

**Analyse de variance multiniveau pour la séparation de l'influence des différentes composantes optiques d'un analiseur avec cuvettes utilisables q'une seule fois**

L'imprécision optique de mesures d'absorption d'équilibre dans un analiseur centrifugal avec cuvettes à jeter a été separé en quatre parties par une analyse de variance multiniveau. Le coéfficient de variance global pour mesures d'absorption 2052 sur trois roteurs tirés sur trois lots différents était de 3.4% ($\bar{x}$ = 0.4144). La partie principale de la variance (68.6%) était due à la variation entre les lots. La variation entre les roteurs était de 10.2% du total, la variance entre cuvettes dans un roteur

**Vielstufen Varianz-Analyse zur Unterteilung der Bestandteile der optischen Ungenauigkeit in einem Analysensystem mit wegwerfbaren Kuvetten**

Die optische Ungenauigkeit von Gleichgewichts-Absorptionsmessungen auf einem Zentrifugen-Analysator, der wegwerfbare Kuvetten verwendet, wurde mit einer vielstufen Varianz-Analyse in vier Bestandteile unterteilt. Der Gesamt-Variationskoeffizient für 2052 Absorptionsmessungen auf drei wegwerfbaren Rotoren von je drei Stichproben von Rotoren war 3.4% ($\bar{x}$ = 0.4144). Die meiste Varianz (68.6%) war auf die Variation zwischen den Stichproben zurückzuführen. Variation zwischen cuvette-in-rotor variance was 17.2% of the total, and the variance component attributed to a single photometric reading was 4.0% of the total variance. The standard deviation of a single photometric sampling was calculated to be 2.8 milli absorbance units (CV = 0.7%) at an absorbance of 0.4144.

était de 17.2% du total et la variance due à la mesure photométrique était de 4.0% de la variance totale. L'écart-type d'un échantillon photométrique était de 2.8 unités de milliabsorption (CV = 0.7%) pour une absorption de 0.4144.

den Rotoren war 10.2% des Total, Varianz zwischen den Kuvetten im Rotor war 17.2% des Total, und die Varianzkomponente, die auf eine einzelne photometrische Ablesung zurückzuführen ist, betrug 4.0% der totalen Varianz. Die Standardabweichung einer einzelnen photometrischen Messung wurde zu 2.8 milli-Absorptionseinheiten (CV = 0.7%) berechnet bei einer Absorption von 0.4144.

### Page 27

**The clinical applicability of a new automatic sugar analyser**

K. Dörner, G. Georgi, G. Liebezeit and R. Dawson

A new chromatographic system with Nanochrom II as the reagent has been tested for the determination of sugars in urine, serum and spinal fluid. The analytical precision (as expressed by a coefficient of variation of 1.2 - 5.4%) and reproducibility (expressed by recoveries of 84.2 - 99.1%) were considered to be satisfactory for routine use. The detection limit ranged from 0.38 to 0.02 mmoles/l and may easily be decreased to lower concentrations. Column chromatography was comparable or superior to all other conventional methods in all aspects investigated. Samples were analysed after ultrafiltration without further pretreatment. The interference of certain drugs was tested.

**L'application clinique d'un nouvel analysateur automatique pour sucres**

Un nouveau système chromatographique avec Nanochrom II comme réactif a été testé pour la détermination de sucres dans l'urine, le sérum et le liquide spinal. La précision analytique (exprimé comme coéfficient de variation de 1.2 – 5.4%) et la reproductibilité (exprimé par les valeurs retrouvés de 84.2 – 99.1%) sont considérés satisfaisants pour la routine. La limite de détection variait entre 0.38 et 0.02 mmoles/l et peut facilement être réduite à des concentrations inférieures. La chromatographie par colonne était comparable ou supérieure à toutes les autres méthodes conventionelles pour tous les aspects étudiés. Les échantillons ont été analysés aprés ultrafiltration sans autre prétraitement. L'interférence d'autres substances à été testée.

**Die klinische Anwendbarkeit eines neuen automatischen Zucker-Analysators**

Es wurde ein neues chromatographisches System, das Nanochrom II als Reagenz verwendet, für die Bestimmung von Zucker in Urin, Serum und Liquor getestet. Die analytische Präzision (ausgedrückt durch einen Variationskoeffizienten von 1.2 – 5.4%) und Reproduzierbarkeit (ausgedrückt durch wiedergefundene Werte von 84.2 – 99.1%) werden als für den Routinegebrauch genügend betrachtet. Die Detektionsgrenze variierte von 0.38 – 0.02 mMol/l und kann leicht zu tieferen Konzentrationen erweitert werden. Kolonnenchromatographie war in allen untersuchten Aspekten allen anderen konventionellen Methoden vergleichbar oder überlegen. Proben wurden nach Ultrafiltration ohne jede weitere Vorbehandlung analysiert. Ferner wurde die Interferenz gewisser Drogen getestet.

### Page 30

**Automated titrations: the use of automated multiple flow injection analysis for the titration of discrete samples**

K.K. Stewart and A.G. Rosenfeld

A general approach and apparatus are described which permit the automated titration of discrete samples in automated multiple flow injection analysis (AMFI). The system contains a new inexpensive exponential gradient chamber and has been used with automated feedback for the sequential titration of samples of widely varying solute concentrations. Sixty titrations per hour can be performed under normal operating conditions.

**Titrages automatiques: utilisation d'un système automatique par multiple "flow injection" pour le titrage d'échantillons discrets.**

Une étude générale ainsi q'un appareil sont décrits pour le titrage automatique d'échantillons discrets par une automatique et multiple "flow injection analysis" (AMFI). Le système contient une nouvelle chambre à gradient exponentiel bonmarchée et a été utilisée avec "feedback" automatique pour le titrage d'échantillons de concentrations très variables. Soixante titrages peuvent être exécutés par heure sous conditions d'opération normales.

**Automatische Titrationen: die Verwendung der automatischen Vielfach-Einspritzanalyse für die Titration diskreter Proben**

Es wird ein allgemeines Konzept und eine Apparatur beschrieben, welche die automatische Titration diskreter Proben in einem automatischen Vielfach-Einspritzanalysator (automated multiple flow injection) erlaubt. Das System enthält eine neue, billige exponentielle Gradientenkammer und wurde mit automatischer Rückkopplung für die sequentielle Titration von Proben mit breit streuendem Konzentrationsbereich verwendet. Sechzig Titrationen pro Stunde können unter normalen Betriebsbedingungen durchgeführt werden.

### Page 33

**Evaluation of the BCL clinicon chemcode system**

A.D. Di Michiel, R.J. Wood, H.G. Worth and D.G. Williams

The BCL Clinicon Chemcode, a microprocessor-controlled photometric system designed for endpoint, kinetic and fixed point kinetic modes for analysis of blood was evaluated. Glucose, urea and calcium were measured in the endpoint mode, aspartate aminotransferase, alkaline phosphatase in the kinetic mode and $\gamma$-glutamyl transferase in both the kinetic and fixed point kinetic modes. Overall, there was good correlation with methods in routine use and precision was acceptable, except for urea using an enzymic-UV method. An electrical safety audit was also performed. The instrument proved to be easy to use and gave no trouble. It should be useful for paediatric and emergency work, and as a back up for other clinical analysers.

**Evaluation du système Clinicon Chemcode de BCL**

Le BCL Clinicon Chemcode, un système photométrique controllé par microprocesseur et prévu pour l'analyse de sang avec les principes à point final, cinétiques et cinétiques à point fixe a été évalué. La glucose, l'uréa et le calcium ont été mesurés dans le mode à point final, l'aspartate aminotransférase et la phosphatase alcaline dans le mode cinétique et la gammaglutamyle transférase avec le mode cinétique et le mode cinétique à point fixe. La corrélation avec les méthodes de routine ainsi que la précision étaient acceptables, excepté pour l'uréa en utilisant une méthode enzymatique/ UV. La sécurité électrique a également été testée. L'instrument était facile à utiliser et n'a donné aucun problème. Il devrait être utile pour le travail pédiatrique et d'urgence et comme deuxième appareil à coté d'un analyseur clinique.

**Beurteilung des BCL "Clinicon chemcode" System**

Es wurde der BCL "Clinicon Chemcode", ein durch einen Mikroprozessor gesteuertes photometrisches System für Endpunkts-, kinetische und kinetische Fixpunkt-Analyse von Blut beurteilt. Glucose, Harnstoff und Kalzium wurden mit der Endpunktsanalyse bestimmt, Aspartat-Aminotransferase, alkalische Phosphatase mit der kinetischen Methode und Gamma-Glutamyl Transferase mit der kinetischen und der kinetischen Fixpunkt-Methode bestimmt. Im gesamten wurde eine gute Korrelation gefunden mit Methoden, die im Routinegebrauch stehen, und die Präzision war, mit Ausnahme von Harnstoff mit der enzymischen UV-Methode, genügend. Eine Ueberprüfung der elektrischen Sicherheitsaspekte wurde ebenfalls durchgeführt. Das Instrument erwies sich als einfach zu gebrauchen und bereitete keine Schwierigkeiten. Es sollte sich als nützlich für pädriatische und Notfall-Arbeit sowie als Reserveinstrument zu anderen klinischen Analysatoren erweisen.

